# Effects of different weaning times on the stress response and the intestinal microbiota composition of female forest musk deer *(Moschus berezovskii)* and their fawns

**DOI:** 10.1371/journal.pone.0276542

**Published:** 2022-10-20

**Authors:** Yimeng Li, Minghui Shi, Baofeng Zhang, Jiahui Wu, Yichen Wang, Mengqi Li, Yining Wu, Xin Hu, Defu Hu, Zhixin Huang, Torsten Wronski

**Affiliations:** 1 Beijing Museum of Natural History, Beijing, China; 2 School of Ecology and Nature Conservation, Beijing Forestry University, Beijing, China; 3 Beijing Key Laboratory of Captive Wildlife Technology, Beijing Zoo, Beijing, China; 4 Zhangzhou Pien Tze Huang Pharmaceutical Co., Ltd, Zhangzhou City, Fujian Province, China; 5 School of Natural Sciences and Psychology, Liverpool John Moores University, Liverpool, United Kingdom; Universidade Federal do Rio de Janeiro, BRAZIL

## Abstract

The effects of mother-infant separation (i.e., weaning) on the physiology, psychology and nutrition of mammalian infants have attracted much attention. Forest musk deer (FMD) is a first-class protected species in China and listed endangered in the IUCN Red List. The captive breeding population is not only an important source for restocking of wild resources, but also a necessary way to supply the market with legal musk. So far, there is no scientific basis for the appropriate separation time of FMD females and their infants. Therefore, we used metagenome sequencing and enzyme-linked immunosorbent assays to study changes in the fecal cortisol concentration, as well as the intestinal microbiome composition and function of females and fawns at three different separation times, i.e., after 80 days, 90 days and 100 days. The results showed that the increment of the cortisol concentration in female FMD increased with increasing lactation time. The increment of cortisol concentration in infant FMD was highest in the 80 days weaning group, but there was no significant difference between the 90 days and the 100 days separation time. Based on the annotation results of COG, KEGG and CAZy databases, the abundance of different functions annotated by the intestinal microbiome of mothers and fawns of the 90 days weaning group changed slightly after separation. Based on the above results, the separation of mother and infant FMD is recommended after 90 days, i.e., the separation time that triggered the lowest rate of weaning stress and that supported a relatively stable gastro-intestinal physiology.

## Introduction

Since times immemorial, forest musk deer (*Moschus berezovskii*; hereafter abbreviated FMD) were hunted for their musk, a substance used in Chinese traditional medicine and which is thus of high economic value. The uncontrolled off-take led to a dramatic decline of wild populations in recent decades [1, 2], arousing the importance of captive breeding programs and to legally satisfy an ever-increasing demand for musk [3–6]. Currently, the FMD is classified as ‘endangered’ by the IUCN Red List and represents the major captive stock held on private and governmental musk deer farms in China. Severe timidity and petrified flight responses make it difficult to breed FMD in captivity [7–9]. Moreover, species-inappropriate health and welfare conditions often lead to behavioral disorders, diseases or even mortalities [10].

For young mammals, the mother-infant separation or weaning is a complex process, often accompanied by physiological, behavioral and nutritional changes [11]. In captivity, FMD fawns are usually separated from their mothers at the age of three months and transferred to a single-individual breeding pen [12]. FMD fawns that were detached from their mothers and their familiar environment, no longer suckle easily digestible breast milk, and their daily life changes from maternal dependence to complete autonomy. FMD fawns show a strong behavioral reaction during the first days after weaning, including stereotypic behavior such as walking back and forth, nervous sniffing, agitated running, or trying to jump across the wall fence [13]. Moreover, FMD fawns significantly reduce their food intake during the first few days after weaning, resulting in weight loss and dehydration. Following a period of seven days after weaning, fawns start feeding again and swiftly regain body weight [13]. These observations correspond to findings of Li et al. [14], who showed that the mother-infant separation is a strong stressor for fawns, which is manifested by a sharp rise of the fecal cortisol concentration. This weaning stress is commonly reported from other captive mammals such as foals, calves, and piglets [15–17], and can easily lead to an imbalanced function of the gastrointestinal tract, causing diarrhea (or other diseases) and changes to the composition of intestinal microbiota [18].

The mortality of young FMD in captivity can reach 27% to the age of three months, i.e., the period prior and immediately after weaning [19]. In subsequent months the survival rate gradually increases, indicating that mortalities occur mainly during early infancy. The data further suggest that weaning stress has a lasting negative impact on FMD well into adulthood, and that management measures should be put in place to avoid or mitigate the negative impact of weaning stress. Previous research on the weaning of FMD fawns focused on the effects of weaning time on the behavior of fawns, the weight change in mothers and fawns after weaning or the effect of weaning time on the mother’s reproductive rate [13, 20]. No study has yet investigated the impact of different weaning times on the stress level of fawns or on changes in the intestinal microbiota composition of FMD females and their fawns. Moreover, until to date no scientific study has attempted to identify the suitable weaning time, or to define guidelines to standardize the reproductive management of captive FMD among breeding centers. This study therefore aims to identify the appropriate weaning age, i.e., the best time to separate mothers from their fawns. For this, we analyzed changes in the stress response of fawns by measuring the fecal cortisol concentration before and after three different weaning ages, and we determined the intestinal microbiota composition and function following the same time schedule. The data will enable us to identify the least distressing weaning age for both, mothers and their fawns, and it will help to reduce the adverse effects of weaning stress in captive FMD fawns and thus enhance their survival chances.

## Materials and methods

### Ethics statement

This study was carried out in accordance with the recommendations of the Institute of Animal Care and the Ethics Committee of Beijing Forestry University. The Ethics Committee of Beijing Forestry University also approved the experimental protocol, while the collection of fecal samples was endorsed by the Pien Tze Huang Forest Musk Deer Farm.

### Study area

The study was carried out in the Pien Tze Huang Forest Musk Deer farm, in Huangniupu Town, near Baoji City in Shaanxi Province (E106°47’9", N34°14’46"). The breeding center is located at an altitude of 1,200 to 1,600 m, and the mean annual temperature, precipitation and humidity are 11.4°C, 613.2 mm and 74%, respectively. The farm is situated in the natural habitat of FMD in a remote valley far away from roads and villages. It comprises of 24 breeding compartments, each containing six separate single-individual breeding pens (3.5 × 2 m^2^) and a communal activity space (10 × 7.5 m^2^). The stocking mode is one adult male plus five reproductive females in each compartment. Natural food from the surrounding forest (mainly mulberry leaves), mixed feed-concentrates and drinking water were supplied ad libitum by the management of the farm.

### Sample collection

In captivity, female FMD usually give birth from early June to late July, breast-feeding the fawn until late September or early October [3, 4]. By the end of October, the females enter already the new breeding cycle, so that suckling the fawn for too long would collide with the new breeding sequence and the first post-partum estrus. FMD breeding managers thus prefer a rather short weaning age of maximum three months (approx. 90 days). Moreover, this timing corresponds to the age at which the composition of the fawn’s intestinal microbiota reaches stability, i.e., about 80 days after birth [14]. We therefore defined three different separation times: six female and their infants were separated after 80 days (group A), six after 90 days (group B) and another six after 100 days (group C; S1 Table). Mothers and fawns were sampled one week prior to separation (A1, B1, C1) as well as one week after (A2, B2, C2). To avoid strong stress responses immediately after weaning, human disturbance, e.g., the collection of feces from breeding pens, was suspended during the first three days after weaning. All individuals sampled were healthy and not administered any antibiotic or anthelmintic drugs, and all fawns were delivered through vaginal birth. The evening prior to sampling, the enclosure was cleaned thoroughly, and fresh feces of mother and fawn were collected before dawn of the following day. Fecal pellets of females and fawns were distinguished by different sizes, i.e., adult pellets are obviously larger than those of fawns. Fecal samples were collected into sterile centrifuge tubes, sealed, labelled, and preserved at −80°C until further processed in the laboratory.

### Quantitation of the water content in fecal samples

Fecal samples were milled and approximately 0.5 g transferred into sterile centrifuge tubes. Subsequently, the weight of the empty tube (G2) and weight of the filled tube were established (G1). Sampling tubes were then dried at 65°C in a drying oven for eight hours until the weight of each tube remained unchanged (G3). The percentage water content of each fecal sample was then calculated as: P_water_ = (G2 − G3) / (G2 − G1).

### Determination of fecal cortisol concentration

Feces (approximately 0.5 g) were transferred into 10 mL sterile centrifuge tubes. Subsequently, 5 mL of 90% (v/v) ethanol were added and agitated for one minute before being centrifuged at 2,500 rpms for 20 minutes. The supernatant was collected and another 5 mL of 90% (v/v) ethanol were added before being agitated for 20 minutes and again centrifuged at 2,500 rpms for 20 minutes. The supernatants from the two extraction steps were pooled together and the ethanol was evaporated in a 60°C water bath. Later, one mL of phosphate buffer solution (PBS) was added, and the tubes were vibrated for five minutes using ultrasonic instrumentation. Samples were then frozen at −20°C until further processed. Since FMD are phylogenetically close to bovids [21, 22], fecal cortisol was measured using Bovine ELISA kits from Reagent Genie Ltd (Ireland). Three replicates were run on each sample.

### DNA extraction and sequencing

QIAamp Fast DNA Stool Mini Kit (QIAGEN, Hilden, Germany) was used to extract fecal DNA of female and infant FMD. The detailed extraction process was conducted followed manufacturer’s instructions. The integrity of DNA was tested by 1% agarose gel electrophoresis. The concentration of DNA was assessed using a Qubit^TM^ dsDNA HS Assay Kit (Life Technologies, Carlsbad, USA). DNA was fragmented by Covaris M220 to generate an average size of approximately 400 bp fragments. Subsequently, the paired-end library was constructed using the NEXTflex Rapid DNA-Seq kit (Bioo Scientific, Austin, TX, USA). Paired-end sequencing was conducted by Shanghai Meiji Biomedical Technology Co., Ltd. (Shanghai, China) using the Illumina NovaSeq platform (Illumina Inc., San Diego, CA, USA).

### Bioinformatic analysis

Representative sequences of a non-redundant gene catalogue were aligned with the NCBI NR database using an e-value cut-off of 1e-5 in Diamond (version 0.8.35) for taxonomic annotations. Diamond (version 0.8.35) was also used to compare the amino acid sequences with the EggNOG (evolutionary genealogy of genes: Non-supervised Orthologous Groups) Database to obtain the COG (Cluster of Orthologous Groups of proteins) function corresponding to the respective gene. Subsequently, the COG abundance was calculated as the sum of the gene abundances corresponding to the COG and compared with KEGG (Kyoto Encyclopedia of Genes and Genomes) database to obtain the KEGG function. Hmmscan was used to compare the amino acid sequence to the CAZy (Carbohydrate-Active enzymes) database and to obtain an annotation of the carbohydrate-active enzyme. The abundance of the carbohydrate-active enzyme was calculated as the sum of the gene abundances. To test for differences in the cortisol concentration between groups, one-way analysis of variance (ANOVA) were applied using SPSS (Version 19.0). To test for differences in bacteria abundance before and after separation, either a Student’s t-test (for normally distributed data) or a Mann–Whitney U-test (for non-normally distributed data) were applied using SPSS (Version 19.0).

## Results

### Changes of fecal cortisol concentration before and after separation of female and infant FMD at different separation times

**Fig 1:**
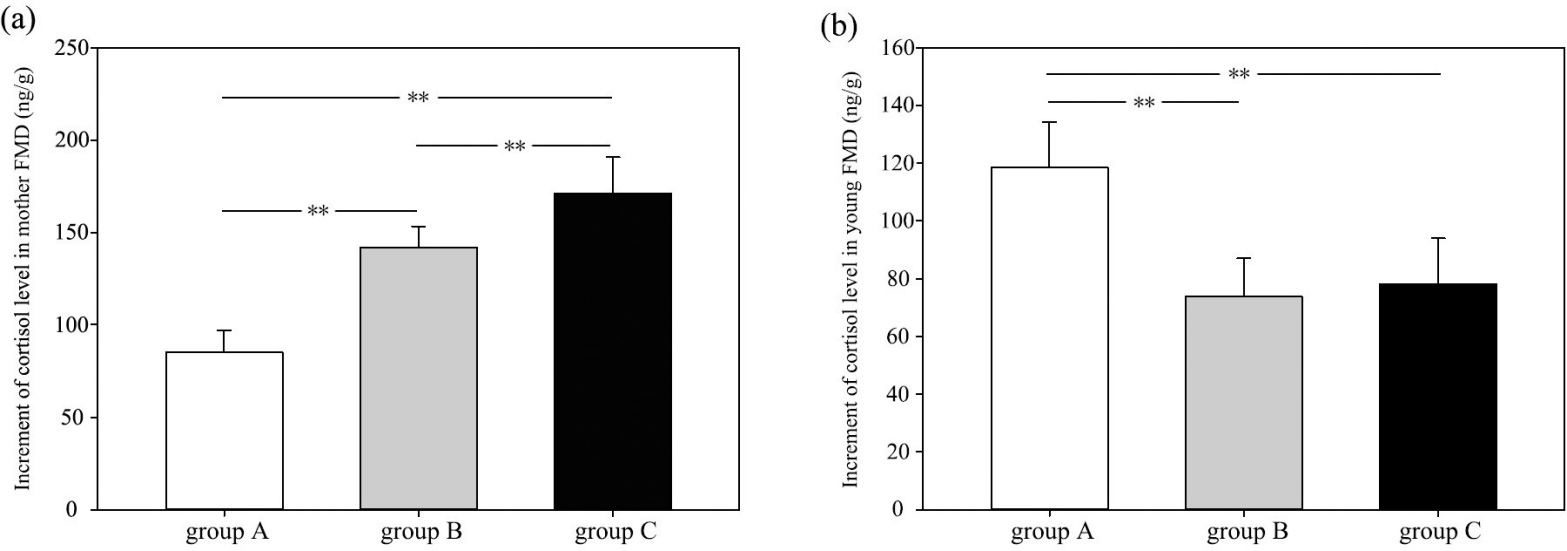
Increment of the fecal cortisol concentration of female (a) and infant FMD (b) before and after separation at different separation times (group A: 80 days, group B: 90 days, group C: 100 days).

In group A, B and C, the average increment of the fecal cortisol concentration in female FMD after separation was 85.13 ± 11.71 ng/g feces, 142.00 ± 11.40 ng/g feces, and 171.11 ± 19.57 ng/g feces, respectively (Fig 1). The increment of group C was significantly higher than that of group A and B (one-way ANOVA, *P* < 0.01). The average increment of the fecal cortisol concentration in fawns after separation was 118.57 ± 15.82 ng/g feces, 73.85 ± 13.28 ng/g feces, and 78.20 ± 15.82 ng/g feces, respectively. The increment of the cortisol concentration in group A was significantly higher than that in group B and C (one-way ANOVA, *P* < 0.01), but there was no significant difference between group B and C (one-way ANOVA, *P* > 0.05).

### Composition of intestinal microbiota before and after separation of female and infant FMD at different separation times

At the phylum level (top five; Table 1) in female FMD of group A, the abundance of Firmicutes and Bacteroidetes increased significantly after separation (*P* < 0.05), while the abundance of Actinobacteria significantly decreased (*P* < 0.05). In females of group B, the abundance of Firmicutes increased significantly after separation (*P* < 0.05), whereas the abundance of Bacteroidetes and Actinobacteria significantly decreased (*P* < 0.05). In females of group C, the abundance of Firmicutes and Bacteroidetes decreased significantly after separation from the mother (*P* < 0.05), while the abundance of Actinobacteria increased significantly (*P* < 0.05). At the genus level (top five; Table 2) in female FMD of group A, the abundance of *Bacteroides* and *Ruminococcus* increased significantly after separation (*P* < 0.05). By contrast in female FMD of group B, no significant difference in the abundance of *Bacteroides* before and after separation was observed (*P* > 0.05), but the abundance of *Ruminococcus* increased significantly (*P* < 0.05). In female FMD of group C, the abundance of *Bacteroides* and *Ruminococcus* decreased significantly after separation (P < 0.05), but no genus showed increased abundance.

**Table 1:** Differences in the five most abundant phyla of intestinal microbiota in female and infant FMD at different separation times (A: 80 days, B: 90 days, C: 100 days) as well as before (1) and after (2) separation.

	Firmicutes	Bacteroidetes	Actinobacteria	Proteobacteria	Verrucomicrobia
A1-female	65.93%±4.03%	13.15%±1.09%	10.30%±3.63%	1.88%±0.17%	0.71%±0.31%
A2-female	73.68%±3.06%	16.2%±1.49%	5.91%±2.70%	1.30%±0.21%	0.61%±0.36%
*P value*	*P<0*.*05*	*P<0*.*05*	*P*<0.05	*P*<0.05	*P*>0.05
B1-female	61.64%±6.10%	20.32%±1.80%	7.45%±1.68%	2.08%±0.41%	1.66%±0.37%
B2-female	68.34%±4.71%	17.50%±2.24%	3.19%±0.85%	1.64%±0.26%	1.30%±0.25%
*P value*	*P*<0.05	*P*<0.05	*P*<0.05	*P*<0.05	*P*<0.05
C1-female	67.01%±2.47%	15.47%±3.42%	10.34%±2.78%	1.56%±0.52%	1.01%±0.39%
C2-female	63.13%±1.85%	11.24%±2.76%	12.61%±2.52%	2.49%±0.37%	0.28%±0.12%
*P value*	*P<0*.*05*	*P<0*.*05*	*P<0*.*05*	*P<0*.*05*	*P<0*.*05*
A1-infant	62.97%±12.65%	14.56%±4.78%	12.96%±2.95%	2.23%±1.02%	1.60%±0.88%
A2-infant	76.16%±10.33%	13.53%±5.29%	3.98%±1.21%	2.04%±1.54%	0.27%±0.18%
*P value*	*P<0*.*05*	*P<0*.*05*	*P<0*.*05*	*P<0*.*05*	*P<0*.*05*
B2-infant	69.93%±8.72%	16.25%±3.77%	5.05%±2.01%	1.82%±0.63%	0.92%±0.34%
B2-infant	69.97%±10.16%	19.13%±3.65%	3.34%±1.68%	1.91%±0.77%	1.04%±0.29%
*P value*	*P*>0.05	*P*<0.05	*P*<0.05	*P*<0.05	*P*<0.05
C1-infant	68.87%±7.45%	16.21%±3.85%	7.41%±2.11%	1.78%±0.54%	0.60%±0.25%
C2-infant	64.21%±4.32	12.08%±4.07%	7.95%±3.43%	2.36%±1.36%	8.08%±2.32%
*P value*	*P*<0.05	*P*<0.05	*P*>0.05	*P*<0.05	*P*<0.05

**Table 2:** Differences in the five most abundant genera of intestinal microbiota in female and infant FMD at different separation times (A: 80 days, B: 90 days, C: 100 days) as well as before (1) and after (2) separation.

	*Bacteroides*	*Alistipes*	*Ruminococcus*	*Prevotella*	*Faecalibacterium*
A1-female	5.25%±2.77%	4.27%±1.72%	3.42%±1.45%	1.26%±0.49%	2.63%±1.33%
A2-female	8.63%±2.15%	5.36%±3.22%	4.95%±1.28%	1.72%±0.73	2.48%±0.87%
*P value*	*P<0*.*05*	*P>0*.*05*	*P<0*.*05*	*P<0*.*05*	*P*>0.05
B1-female	8.99%±3.56%	6.45%±2.14%	3.63%±1.76%	2.80%±1.58%	1.66%±0.42%
B2-female	8.95%±3.10%	3.26%±1.98%	4.93%±2.01%	3.67%±1.07%	1.30%±0.87%
*P value*	*P>0*.*05*	*P<0*.*05*	*P<0*.*05*	*P<0*.*05*	*P>0*.*05*
C1-female	6.80%±2.32%	4.28%±1.90%	4.11%±1.21%	1.67%±0.41%	2.22%±0.76%
C2-female	4.58%±1.77%	3.50%±1.52%	3.37%±1.54%	1.10%±0.27%	1.89%±0.51%
*P value*	*P<0*.*05*	*P>0*.*05*	*P*<0.05	*P>0*.*05*	*P>0*.*05*
A1-infant	6.20%±3.24%	4.28%±0.75%	3.69%±1.72%	1.44%±0.56%	1.53%±0.82%
A2-infant	6.25%±2.80%	3.56%±0.32%	5.08%±1.21%	1.32%±0.42%	1.69%±0.54%
*P value*	*P*>0.05	*P>0*.*05*	*P*<0.05	*P*>0.05	*P*>0.05
B2-infant	6.66%±3.02%	4.77%±0.33%	3.60%±1.01%	1.81%±0.33%	3.95%±0.87%
B2-infant	9.70%±2.88%	3.40%±0.41%	4.48%±0.89%	2.64%±0.47%	2.14%±1.12%
*P value*	*P*<0.05	*P<0*.*05*	*P<0*.*05*	*P<0*.*05*	*P*<0.05
C1-infant	7.51%±2.62%	3.46%±0.82%	4.00%±2.62%	1.67%±0.52%	2.57%±0.53%
C2-infant	5.11%±2.60%	3.18%±0.55%	3.95%±1.74%	1.32%±0.29%	1.78%±0.29%
*P value*	*P*<0.05	*P>0*.*05*	*P*>0.05	*P>0*.*05*	*P<0*.*05*

At the phylum level (top five; Table 1) in infant FMD of group A, the abundance of Firmicutes increased significantly after separation (*P* < 0.05), while the abundance of Bacteroidetes and Actinobacteria significantly decreased after separation (*P* < 0.05). In fawns of group B, the abundance of Firmicutes showed no significant difference before and after separation (*P* > 0.05), but the abundance of Bacteroidetes increased significantly (*P* < 0.05), whereas the abundance of Actinobacteria significantly decreased after separation (*P* < 0.05). In fawns of group C, the abundance of Firmicutes and Bacteroidetes significantly decreased after separation (*P* < 0.05), while the abundance of Actinobacteria significantly increased (*P* < 0.05). At the genus level (top five; Table 2) in fawns of group A, no significant difference in the abundance of *Bacteroides* before and after separation was unraveled (*P* > 0.05), but the abundance of *Ruminococcus* significantly increased after separation (*P* < 0.05). For fawns of group B, the abundance of *Bacteroides* and *Ruminococcus* increased significantly after separation (*P* < 0.05), whereas in fawns of group C the abundance of *Bacteroides* significantly decreased after separation (*P* < 0.05). For *Ruminococcus* no significant difference was unraveled in fawns of group C before and after separation (*P* > 0.05).

### Functional differences of intestinal microbiota before and after separation of female and infant FMD at different separation times

**Fig 2:**
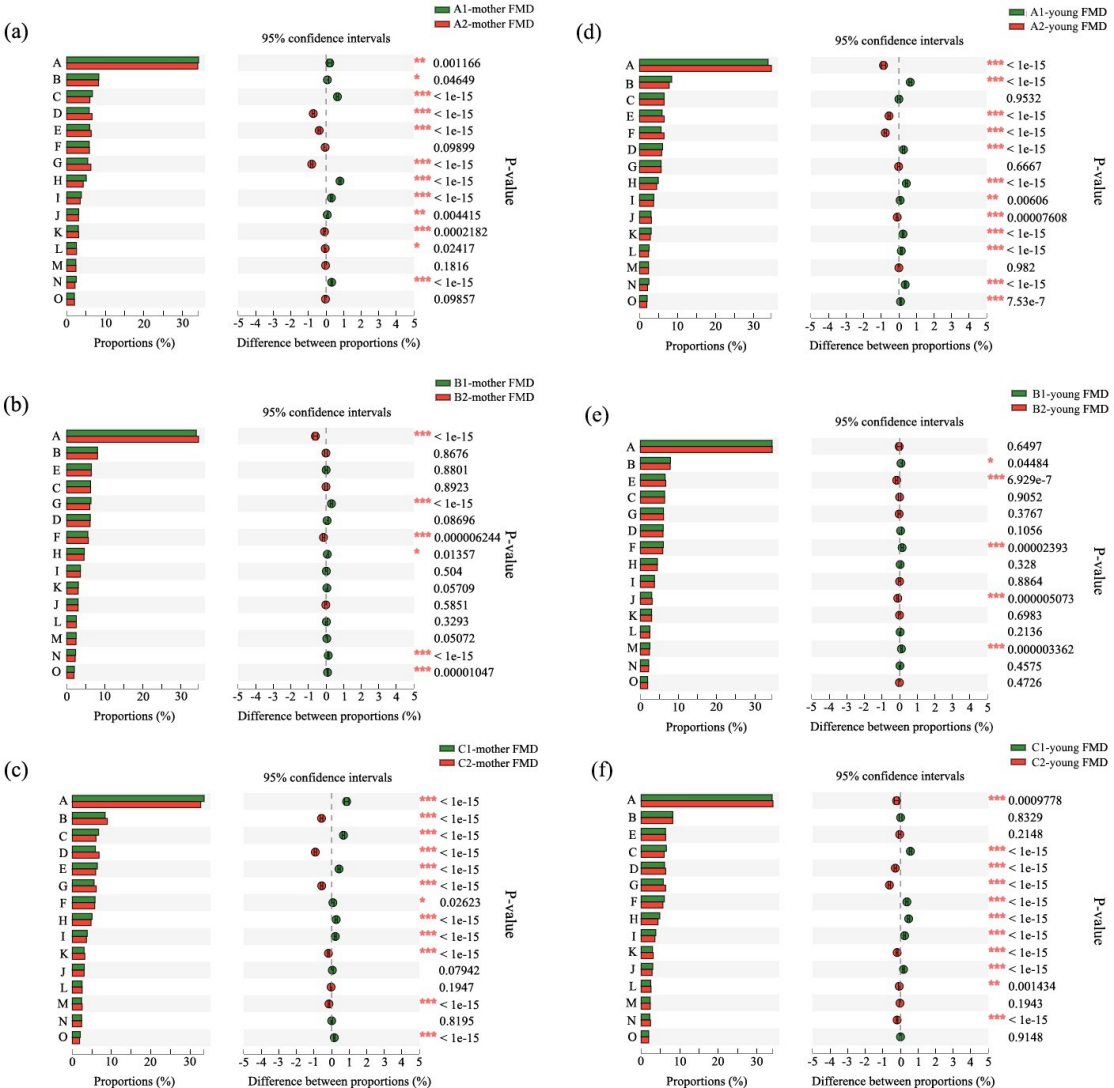
Comparison of functional differences before and after separation of female (a-c) and infant FMD (d-f) based on the COG database at different separation times (a, d: 80 days; b, e: 90 days; c, f: 100 days). A: Function unknown; B: Replication, recombination and repair; C: Amino acid metabolism transport and metabolism; D: Translation, ribosomal structure and biogenesis; E: Carbohydrate transport and metabolism; F: Transcription; G: Cell wall/membrane/envelope biogenesis; H: Energy production and conversion; I: Inorganic ion transport and metabolism; J: Signal transduction mechanisms; K: Posttranslational modification, protein turnover, chaperones; L: Nucleotide transport and metabolism; M: Defense mechanisms; N: Coenzyme transport and metabolism; O: Lipid transport and metabolism.

**Fig 3:**
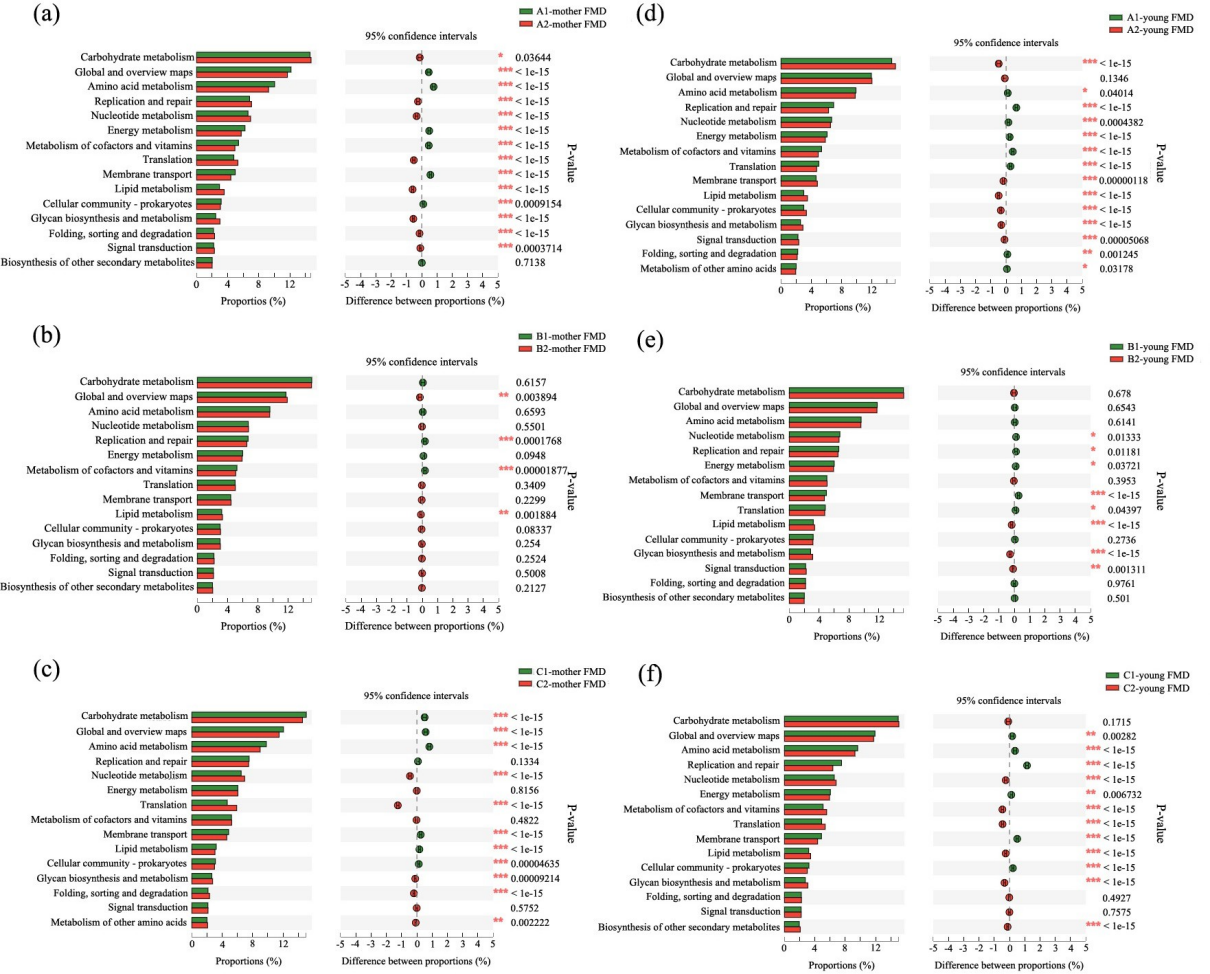
Comparison of functional differences before and after separation of female (a-c) and infant FMD (d-f) based on the KEGG database at different separation times (a, d: 80 days; b, e: 90 days; c, f: 100 days).

**Fig 4:**
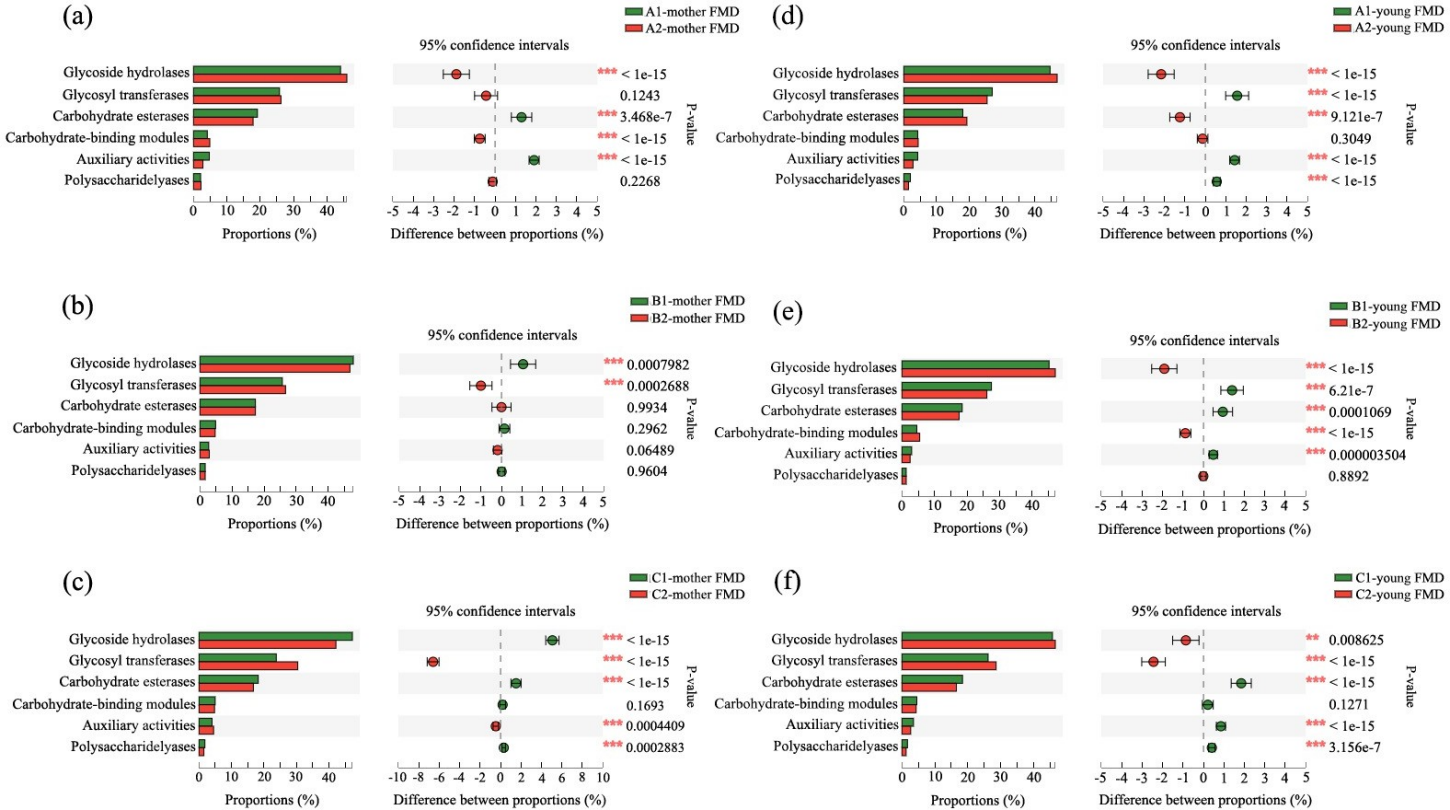
Comparison of functional differences before and after separation of female (a-c) and infant FMD (d-f) based on the CAZy database at different separation times (a, d: 80 days; b, e: 90 days; c, f: 100 days).

Statistical charts of the number of annotated genes in the COG, KEGG and CAZy databases were based on the annotation results of unigenes and were depicted in Figs 2–4. Based on the annotation of the COG database (a database for the analysis of protein functions) for female FMD of group A (Fig 2), the relative abundance of carbohydrate transport and metabolism function was significantly higher than that before separation (*P* < 0.05). By contrast, no significant difference was observed in the relative abundance of carbohydrate transport and metabolism function before and after separation in group B (*P* > 0.05). In group C, however, the relative abundance of carbohydrate transport and metabolism function was significantly lower than that before separation (*P* < 0.05). In infant FMD of group A and B, the relative abundance of carbohydrate transport and metabolic function was significantly higher than that before separation (*P* < 0.05), and there was no significant difference in the relative abundance of carbohydrate transport and metabolic function before and after separation in group C (*P* > 0.05). When comparing the changes of intestinal microbiota function in female and infant FMD before and after weaning at three different separation times, it can be summarized that in female and infant FMD of group B the abundance of functional groups remained stable after separation, whereas that of group A and C showed significant differences.

KEGG is a database of biological systems that integrates genomic, chemical and systemic functional information. Based on the annotation of the KEGG database, the functional categories with high abundance at the second level were analyzed (Fig 3), among which the carbohydrate metabolism is the functional category with the highest abundance. In female FMD of group A, the relative abundance of carbohydrate metabolism was significantly higher than that before separation (*P* < 0.05), while in group B, the relative abundance of carbohydrate metabolism showed no significant difference between before and after separation (*P* > 0.05). In group C, the relative abundance of carbohydrate metabolism was significantly lower than that before separation (*P* < 0.05). In infant FMD of group A, the relative abundance of carbohydrate metabolism was significantly higher than that before separation (*P* < 0.05), whereas no significant difference in the carbohydrate metabolism was unraveled in group B and C (*P* > 0.05). When comparing the changes of intestinal microbiota function in female and infant FMD before and after weaning at three different separation times, it can be summarized that in group B the abundance of functional groups remained stable after separation whereas in group A and C significant differences were observed.

CAZy is a database of enzymes that can synthesize or decompose complex carbohydrates and glycoconjugates. According to the similarity of amino acid sequences in protein domains and their corresponding functions, the database divides carbohydrate active enzymes into six categories. According to the annotation results of the database (Fig 4), the relative abundance of glycoside hydrolases (GHs) is the highest among the six functional categories. In female FMD of group A, the abundance of GHS increased significantly after separation (*P* < 0.05), while the abundance of GHS significantly decreased in group B and C after separation (*P* < 0.05). For infant FMD, the abundance of GHS increased significantly after separation at all separation times, i.e., in each group (*P* < 0.05).

## Discussion

In this study, metagenome sequencing and enzyme-linked immunosorbent assay were used to study changes in the cortisol level, the intestinal microbiota abundance, and their function in female and infant FMD before and after weaning. Our results showed that different separation times had different effects on the physiological condition of mother and fawn.

The analysis of cortisol concentrations showed that female FMD of group A (separated after 80 days) showed the lowest increment after separation and that they were least affected by weaning stress, while female FMD of group C (separated after 100 days) were the most affected group. Because lactation consumes a lot of energy, longer lactation times lead to greater energy losses, and will thus increase the stress level of the mother. Feng et al. [20] explored the relationship between lactation time and the reproductive performance of female FMD and found that mothers with longer lactation time were thinner and more lightweight. Moreover, early separation of mother and fawn could significantly improve the future reproductive performance of females [20]. In infant FMD, the increment of the cortisol concentration of group A was significantly higher than that of group B and C, whereas the difference between group B and C was insignificant. This result corresponds to findings of Li et al. [23], who found the fecal cortisol concentration of piglets, measured at different weaning times, to be significantly higher when weaned after 14 days than in those weaned after 21, 28 or 35 days. This is because, FMD fawns—or other infant mammals—weaned at an earlier time will be separated from the nutritious breast milk and the care of their mothers, resulting in a significant stronger increase in cortisol concentration than in fawns separated later when the mothers care is already reduced and the weaning process has started. Based on the observed changes in the cortisol concentration, the separation of mother and fawn is therefore recommended after 90 days, i.e., the time when the fawn is least affected by the weaning stress.

Firmicutes and Bacteroidetes are two bacteria phyla that contain many species which can decompose cellulose and polysaccharides [24, 25]. With increasing lactation time in females, the abundance of these microorganisms was significantly lower after separation than that observed before separation. *Ruminococcus* another important decomposer of cellulose [26] showed the same pattern, i.e., with increasing lactation time in females, the post-separation abundance was significantly lower than that before separation. These results suggest that longer lactation times will affect the digestive physiology of females by lowering their ability to digest long-chained polysaccharides. In infant FMD—by contrast—the abundance of *Ruminococcus* in group C did not change significantly after separation, while that of group A and B significantly increased. This was because infant FMD, separated after 100 days (group C), had ingested the same type and amount of food (i.e., mulberry leaves) before and after separation. By contrast the intake of leaves by fawns of group A (separated after 80 days) and group B (separated after 90 days) was gradually increasing after separation, resulting in an increased abundance of cellulose digesting bacteria in their gastro-intestinal tract.

Based on the annotation results of the COG and KEGG databases, the abundance of functional categories of intestinal microbiota in female and infant FMD of group B was relatively stable, indicating that the physiological condition of mothers and their fawns was not affected by the separation. By contrast, in group A and C, the abundance of functional categories had changed substantially, suggesting that the separation had a significant impact on the physiological condition of females and fawns. Based on the annotation results obtained from the CAZy database, the types of carbohydrate active enzymes—encoded by the intestinal microbiota of female and infant FMD at different separation times—were generally similar, while their abundance differed significantly. Furthermore, our results on the functional differences of intestinal microbiota showed that the abundance of glycoside hydrolases was highest in all groups, followed by glycosyltransferase, the carbohydrate binding module, carbohydrate esterase and polysaccharide lyase. The lowest abundance was measured for auxiliary oxidoreductase, which was close to carbohydrate active enzymes encoded by intestinal microbiota recorded from sika deer [27].

### Conclusion

In conclusion, the results of this study clearly suggest that female and infant FMD in captivity were least affected by weaning stress when separated after 90 days. Timely weaning of fawns can significantly reduce the adverse effects of weaning stress on mothers as well as their fawns. This result is of great importance for improving the health and welfare conditions of captive FMD, especially that of weaned infants. Our results will help to further improve the management and development of captive FMD populations, not only in China but also in other musk producing countries such as Nepal or Russia. Future key points of research should focus on the elimination of the adverse consequences of weaning stress—in both mothers and their fawns—by testing the effects of the multiple step-by-step separation method or the single sudden mother-infant separation method on the physiology of mothers and fawns.

## Supporting information

S1 Table Individual information table of forest musk deer. (DOCX)

## References

[1] Wemmer C. Deer: Status survey and conservation action plan. 1998, IUCN/SSC Deer Specialist Group, IUCN, Gland.

[2] Wang Y, Harris RB. *Moschus berezovskii*. The IUCN Red List of Threatened Species. Version 2014.1. www.iucnredlist.org. 2008.

[3] Sheng HL, Liu ZX. The musk deer in China. The Shanghai Scientific & Technical Publishers, Shanghai. 2007.

[4] Wu J, Wang W. The musk deer of China. China Forestry Publisher, Beijing; 2007.

[5] Meng X, Yang H, Yang Q, Feng Z, Peng X, Perkins GC. Preliminary findings of behavioral patterns in captive alpine musk deer (*Moschus sifanicus*) and prospects for future conservation. Turk J Vet Anim Sci. 2010;34: 111–117.

[6] Xu K, Bu S, Liang Z, Wang H, Luo C, Zhu C. Research progress in forest musk deer. Heilongjiang Animal Science and Veterinary Medicine. 2014;4: 147–150

[7] Becker BG, Lobato JP. Effect of gentle handling on the reactivity of zebu crossed calves to humans. Appl Anim Behav Sci. 1997;53: 219–224.

[8] Thompson VD. Behavioral response of 12 ungulate species in captivity to the presence of humans. Zoo Biol. 1989;8: 275–297.

[9] Meng X, Zhao C, Hui C, Luan X. Behavioral aspects of captive Alpine musk deer during non-mating season: gender differences and monthly patterns. Asian-Aust J Anim Sci. 2011;24: 707–712.

[10] Morgan KN, Tromborg CT. Sources of stress in captivity. Appl Anim Behav Sci. 2007;102: 262–302.

[11] Waran NK, Clarke N, Famworth M. The effects of weaning on the domestic horse (*Equus caballus*). Appl Anim Behav Sci. 2008;110: 42–57.

[12] Wang YH. Immunoglobulin and physiological stress assays with non-invasive method in captive forest musk deer. PhD. Thesis, Beijing Forestry University. 2011. Available from: http://cdmd.cnki.com.cn/article/cdmd-10022-1011134555.htm

[13] Hu ZQ, Feng XL, Zhao GJ, Chen Q. The Effect of Weaning Ways on Behavior and Growth Performance of Young Forest Musk Deer. Journal of Economic Animal. 2013;17: 214–216.

[14] Li YM, Zhang TX, Shi MH, Zhang BF, Hu X, Xu SH, et al. Characterization of intestinal microbiota and fecal cortisol, T3, and IgA in forest musk deer (*Moschus berezovskii*) from birth to weaning. Integr Zool. 2021;16: 300–312. 33452844 10.1111/1749-4877.12522 PMC8248411

[15] Bruschetta G, Faxio E, Cravana C, Ferlazzo AM. Effects of partial versus complete separation after weaning on plasma serotonin, tryptophan and pituitary-adrenal pattern of Anglo-Arabian foals. Livest Sci. 2017;198: 157–161.

[16] Li Y, Guo Y, Wen ZS, Jiang XM, Ma X, Han XY. Weaning stress perturbs gut microbiome and its metabolic profile in piglets. Sci Rep. 2018;8: 18068. doi: 10.1038/s41598-018-33649-8 30584255 PMC6305375

[17] Arthington JD, Spears JW, Miller DC. The effect of early weaning on feedlot performance and measures of stress in beef calves. J Anim Sci. 2005;83: 933–939. doi: 10.2527/2005.834933x 15753350

[18] Smith F, Clark JE, Overman BL, Tozel CC, Huang JH, Rivier JEF, et al. Early weaning stress impairs development of mucosal barrier function in the porcine intestine. Am J Physiol Gastrointest Liver Physiol. 2010;298: G352–G363. doi: 10.1152/ajpgi.00081.2009 19926814 PMC2838512

[19] Xu ZQ, Xu HF. Population Characteristics and Fawn Survival in Musk Deer (*Moshus moschiferus*). Acta Theriologica Sinica. 2003;23: 17–20.

[20] Feng XL, Zhao GJ, Chen Q, Hu ZQ, Zeng DJ, Zhang CL, et al. The Effect of Lactation Period on Reproductive Performance of Female Forest Musk Deer. Special Wild Economic Animal and Plant Research. 2015;37: 11–13.

[21] Hassanin A, Douzery EJ. Molecular and morphological phylogenies of Ruminantia and the alternative position of the Moschidae. Syst Biol. 2003;52: 206–228. doi: 10.1080/10635150390192726 12746147

[22] Chen L, Qiu Q, Jiang Y, Wang K, Lin ZS, Li ZP, et al. Large-scale ruminant genome sequencing provides insights into their evolution and distinct traits. Science. 2019;364: eaav6202. doi: 10.1126/science.aav6202 31221828

[23] Li LA, Yang JJ, Li Y, Lv L, Xie JJ, Du GM, et al. Effect of weaning age on cortisol release in piglets. Genet Mol Res. 2016;15. doi: 10.4238/gmr.15027693 27173313

[24] Fernando SC, Purvis HT, Najar FZ, Sukharnikov LO, Krehbiel CR, Nagaraja TG, et al. Rumen microbial population dynamics during adaptation to a high-grain diet. Appl Environ Microbiol. 2010;76; 7482–7490. doi: 10.1128/AEM.00388-10 20851965 PMC2976194

[25] Thoetkiattikul H, Mhuantong W, Laothanachareon T, Tangphatsornruang S, Pattarajinda V, Eurwilaichitr L, et al. Comparative Analysis of Microbial Profiles in Cow Rumen Fed with Different Dietary Fiber by Tagged 16S rRNA Gene Pyrosequencing. Curr Microbiol. 2013;67: 130–137. doi: 10.1007/s00284-013-0336-3 23471692

[26] Han XF, Yang YX, Yan HL, Wang XL, Qu L, Chen YL. Rumen bacterial diversity of 80 to 110-day-old goats using 16S rRNA sequencing. PLoS ONE. 2015;10: e0117811. doi: 10.1371/journal.pone.0117811 25700157 PMC4336330

[27] Zhang H, Cong LX, Wei Y, Zhao CX, Liu GW. Effect of different crude fiber source diets on rumen gene function of Sika deer. Chinese Journal of Veterinary Science. 2020;40: 1391–1396.

