# Supplementary material for: Effects of different weaning times on the stress response and the intestinal microbiota composition of female forest musk deer (Moschus berezovskii) and their fawns
Source: PLoS One. 2022 Oct 20;17(10):e0276542. doi: 10.1371/journal.pone.0276542 (PMC9584425; doi:10.1371/journal.pone.0276542)
Supplement: S1 Table — (DOCX) [file pone.0276542.s001.docx]

**Supporting information**

S1 Table. Individual information of 18 captive FMD females and their fawns

| **Mother ID**  **(no. ear tag)** | **Mother age** | **Fawn ID** | **Date of birth of fawn** | **Weaning time** |
| --- | --- | --- | --- | --- |
| 16478 | 5 | YMD1 | 19.06.2020 | 80 days |
| 16504 | 5 | YMD2 | 10.06.2020 | 80 days |
| 17174 | 4 | YMD3 | 10.06.2020 | 80 days |
| 17055 | 4 | YMD4 | 09.06.2020 | 80 days |
| 18340 | 3 | YMD5 | 20.06.2020 | 80 days |
| 23136 | 4 | YMD6 | 15.06.2020 | 80 days |
| 16160 | 5 | YMD7 | 08.06.2020 | 90 days |
| 16294 | 5 | YMD8 | 08.06.2020 | 90 days |
| 16230 | 5 | YMD9 | 08.06.2020 | 90 days |
| 16503 | 5 | YMD10 | 05.06.2020 | 90 days |
| 18330 | 3 | YMD11 | 07.06.2020 | 90 days |
| 18335 | 3 | YMD12 | 08.06.2020 | 90 days |
| 16494 | 5 | YMD13 | 21.05.2020 | 100 days |
| 16485 | 5 | YMD14 | 22.05.2020 | 100 days |
| 17054 | 4 | YMD15 | 21.05.2020 | 100 days |
| 16437 | 5 | YMD16 | 16.05.2020 | 100 days |
| 16301 | 5 | YMD17 | 21.05.2020 | 100 days |
| 16492 | 5 | YMD18 | 20.05.2020 | 100 days |
